# Uterine fibroids may play a protecting role against endometrial carcinoma in Chinese women with gynecological diseases

**DOI:** 10.1042/BSR20200350

**Published:** 2020-07-10

**Authors:** Li Qiao, Lili Xia, Yan Dong, Yajuan Cheng, Hongbao Cao

**Affiliations:** 1 Intensive Care Unit (ICU), The Second People’s Hospital of Lianyungang, Lianyungang, Jiangsu 222000, China; 2 Department of Ultrasound, Tongling People’s Hospital, Tongling, Anhui Province 244000, China; 3 Department of Geriatrics, The Second People’s Hospital of Lianyungang, Lianyungang, Jiangsu Province 222000, China; 4 Department of Psychiatry, First Hospital/First Clinical Medical College of Shanxi Medical University, Taiyuan 030001, China; 5 School of Systems Biology, George Mason University (GMU), Fairfax, VA 22030, U.S.A.

**Keywords:** endometrial carcinoma, HOPX, HTRA3, mega-analysis, PCNA, Uterine fibroids

## Abstract

**Background**: It has been reported that uterine fibroids (UFs) may increase the risk of endometrial carcinoma (EC) with the underlying mechanism largely unknown. Here, we explore whether UF could be an influential factor for EC.

**Methods**: We have collected and analyzed clinical data from 4537 Chinese patients to study the co-incidence of UF and EC. Then, a large-scale literature-based data mining was conducted to identify genes implicated as UF downstream regulating targets and EC upstream regulators. In addition, a meta-analysis has been conducted for each of the EC-specific genes, using six independent UF expression datasets. The meta-analysis results, together with literature-based pathway analysis, were used to explore the potential explanation of the clinical data.

**Results**: Our results showed that the incidence rate of EC in the case of UF was 50.53% lower than without UF, which suggested a protective role of UF in EC patients. The meta-analysis identified three significantly overexpressed genes (*HTRA3, HOPX*, and *PCNA*) in the case of UF, which were implicated as EC inhibitors in the pathway analysis. Multiple linear regression (MLR) analysis showed that, compared with UF, aging might be a stronger influential factor for EC.

**Conclusion**: Among women with gynecological diseases, UFs may play a protecting role against EC in the Chinese population.

## Background

Uterine fibroids (UFs) are common benign uterine smooth muscle tumors that occur in up to 80% women by the age of 50 [1]. Many of these women experience symptoms such as heavy and irregular menstrual bleeding, early pregnancy loss, and infertility. Traditionally believed to be inert masses, fibroids are now known to influence endometrial function at the molecular level.

Endometrial carcinoma (EC) is the most common gynecologic cancer [2] and the sixth most commonly diagnosed cancer in women worldwide [3]. Based on the data of the United States, most endometrial cancers are diagnosed at an early stage (75%), and the reported survival rate is 75% [4]. Some descriptive studies discussed the association between UF and endometrial cancer incidence [5]. However, there are not enough control samples to reach a conclusive result. A recent study [6] focused on investigating the association between UF and endometrial cancer in the Black Women’s Health Study reveals that UF history increased the incidence of EC by up to 42% compared with no UF history [6].

Given the promising development of sequencing technology, several comprehensive genetics and genomics studies were conducted recently to uncover the distinct genetic etiology of EC histological subtypes, i.e., endometrioid, serous, and clear cell [7]. Specific genetic mutations and chromosome abnormalities are reported to be involved in the development of UF [8]. However, genetic studies that cover the overlap samples of EC and UF are still inadequate.

To this end, we present a comprehensive multi-omics study of the UF and EC based on co-incidence samples from the Chinese population. We have collected and jointly analyzed clinical data and expression data. In addition, the literature-based pathway analysis delineated the underlying genetic mechanism of UF’s potential effect on EC. Our study suggested a novel protective role of UF on EC and revealed part of its potential mechanisms at the genetic level.

## Materials and methods

In the present study, the workflow was organized as follows. First, clinical data of 4537 patients were collected and analyzed to study the co-incidence of UF and EC. Then, large-scale literature-based mining was performed to identify genes implicated as UF downstream regulating targets and EC upstream regulators. After that, a meta-analysis was conducted for each of the EC-genes, using six independent UF genes. The meta-analysis results, together with literature-based pathway analysis, were used to explore the potential explanation of the clinical data.

### Clinical data

Clinical data of 4537 patients were collected from Tongling People’s Hospital, where 59 EC cases (age: 53.12 ± 9.04 years) and 1259 UF cases (age: 45.83 ± 6.09 years) were identified, with 12 patients presenting both EC and UF (age: 50.42 ± 8.89 years).

#### Data collection

All patients were enrolled in Tongling People’s Hospital between September 2014 and June 2018. Transabdominal and transvaginal color Doppler examinations were performed before the operation in the hospital. Pathological examinations were used to diagnose endometrial cancer or UFs. Patients with cesarean section complicated with UFs were excluded. The mean age of 4537 patients is 43.98, with standard deviation as 9.64, and the minimum age is 23. For the measurement method, diagnostic criteria, and ultrasound characteristics of EC, please refer to supplementary data UF_EC→UF_EC diagnosis, which is available at http://gousinfo.com/database/Data_Genetic/UF_EC.xlsx.

### Statistical analysis

Bayes’ theorem-based statistical analysis was performed to calculate the conditional probabilities. Specifically, the probability of the incidence of EC under the condition of with/without UF was calculated. The formulas based on Bayes’ theorem describe how to calculate the probability of an event based on its association with another event [9]. Multiple linear regression (MLR) was used to examine the influence on EC of age and co-incidence of other gynecological diseases. All analyses were conducted using the statistical tool of Matlab (version R 2017a).

### EC–gene relation data

Genes negatively/positively regulating EC were extracted from existing literature and analyzed using Pathway Studio (www.pathwaystudio.com) and then were downloaded into a genetic database UF_EC, hosted at http://database.gousinfo.com. The downloadable format of the database in Excel is available at http://gousinfo.com/database/Data_Genetic/UF_EC.xlsx. Besides the list of analyzed genes (UF_EC: EC_Negative, EC_Positive), the supporting references for each disease–gene relation are presented in the database UF_EC (UF_EC: EC_Negative_Ref, EC_Positive_Ref), including titles of the references and the sentences describing identified disease–gene relationships. The information could be used to locate a detailed description of an association of a candidate gene with EC.

### UF expression data meta-analysis

All expression datasets were searched on GEO through a keyword ‘uterine fibroids’ (*n*=78). Then, we applied the following standards as the further filter: (1) the organism was *Homo sapiens*; (2) the data type was RNA expression by array; (3) the sample size was no less than 8; (4) the studies are performed according to case–control design; (5) the dataset and its format files are publically available. Finally, a total of six datasets remained available for the meta-analysis (Table 1).

**Table 1:** UFs datasets used for EC genetic regulator meta-analysis

Study name	GEO ID	#Control/#Case	Country	Sample source
Hoffman et al., 2003	GSE593	5/5	U.S.A.	Uterine leiomyoma
Zavadil et al., 2010	GSE23112	5/5	U.S.A.	Uterine leiomyoma
Chuang et al., 2012	GSE38817	4/4	U.S.A.	Uterine leiomyoma
Maekawa et al., 2013	GSE45188	3/6	Japan	Uterine leiomyoma
Maekawa et al., 2013	GSE45189	3/6	Japan	Uterine leiomyoma
Miyata et al., 2017	GSE68295	3/9	Japan	Uterine leiomyoma and leiomyosarcoma

To note, the selection of the data covers all UF expression array datasets from GEO, which is owned by the National Institute of Health (NIH of U.S.A.). The datasets are publicly available, and no permission or confirmation is needed from any individual investigator(s). Moreover, the datasets extraction had no selection bias concerning publication journals, owner affiliations, and authors. Besides, the original data rather than the processed results of each dataset were used to perform the analysis in the present study, which avoided possible noise caused by the individual data process.

### Meta-analysis models

Meta-analysis is a statistical procedure to combine data from multiple studies. When the treatment effect (or effect size) is consistent from one study to the next, meta-analysis can be used to identify this common effect [10]. Meta-analysis was commonly conducted using a fixed-effect model or a random-effects model [10]. A fixed-effect model is a statistical model in which the model parameters are fixed or non-random quantities. This is in contrast with random-effects models and mixed models in which all or some of the model parameters are considered as random variables. Both the fixed-effects model and the random-effects model were employed here to study the expression level of EC negative and positive regulators (genes) in the case of UF. For a detailed description of the meta-analysis, please refer to supplementary materials UF_EC (Meta-analysis description).

Significant genes were identified according to the following criteria: (1) support by no less than three independent studies; (2) meta-analysis *P*<0.05; and (3) the effect size (log fold change of expression levels, LFC) > 0.59 or < −1. When a gene presents an effect size LFC > 0.59 or < −1 in the meta-analysis, it means that the change of the expression level of the gene increased by more than 50% or decreased by more than 50%. While we present all the meta-analysis results in the UF_EC (EC_Positive_Meta and EC_Negative_Meta), the discussion will be focused on those genes that satisfy the significant criteria.

### MLR analysis

An MLR analysis was employed to study the possible influence of three factors on the gene expression change: sample size, population region, and study date. *P*-values and 95% confidence interval (CI) were reported for each of the factors. The analysis was done in Matlab (R 2017a) with the ‘regress’ statistical analysis package.

### Pathway analysis

The meta-analysis results together with a literature-based functional pathway analysis were conducted with an aim to identify potential biological regulation mechanisms of UF on EC. Specifically, by using Pathway Studio (www.pathwaystudio.com), we first identified UF downstream target genes and EC upstream regulators with polarity. Then we overlay the meta-analysis results to present the genes that demonstrated significance in UF case/control comparison.

## Results

### Clinical data analysis results

In the present study, the total number of women with gynecological diseases was 4537, with 59 (1.30%) diagnosed as EC. To note, the overall incidence rate of women in China is ∼0.063%, which is higher than 0.044% from a previous study on black women in U.S.A. [6]. The 1.30% incidence rate only counts for women with gynecological diseases. The calculation of the EC in the case of UF was presented in (eqns 1 and 2).

(1) $$\text{P}(\text{EC/}\bar{\text{UF}})=\frac{\left(59-12\right)}{\left(4537-1259\right)}\times 100\%=1.43\%$$

(2) $$\text{P}(\text{EC/UF})=\frac{12}{1259}\times 100\%=0.95\%$$

(3) $$\text{EC}\_\text{Ratio}\_\text{Decrease}=\frac{\text{P}(\text{EC/}\bar{\text{UF}})-\text{P}(\text{EC/UF})}{\text{P}(\text{EC/UF})}\times 100\%=50.53\%$$

As shown in (eqn 3), the incidence rate of EC in the case of UF (eqn 2) was lower than without UF (eqn 1) by 50.53% (eqn 3). To note, MLR analysis showed that the incidence of EC is not significant with the co-incidence of other gynecological diseases (*P*-value = 0.12), but is significantly related to aging (*P*-value = 1.00e-13), as shown in Table 2.

**Table 2:** MLR analysis of age and co-incidence of other diseases

	MLR parameters	Other gynecological diseases	Age
**Influence on EC**	β	0.0040	0.0013
	Low limit	−0.0028	0.00093
	Up limit	0.011	0.0016
	*P-value*	0.12	**1.00e-13**
**Influence on UF**	β	0.44	0.0051
	Low limit	0.41	0.0039
	Up limit	0.46	0.0063
	*P-value*	**<1.00e-324**	**<1.00e-324**

The values in bold are the p-values.

### Meta-analysis results

For the meta-analysis of the EC positive regulators (47 genes; see UF_EC: EC_Positive), no gene satisfied the significance criteria, as shown in UF_EC→EC_Positive_Meta. This suggested that, in the six UF datasets, UF did not deactivate any positive regulators of EC.

**Figure 1:**
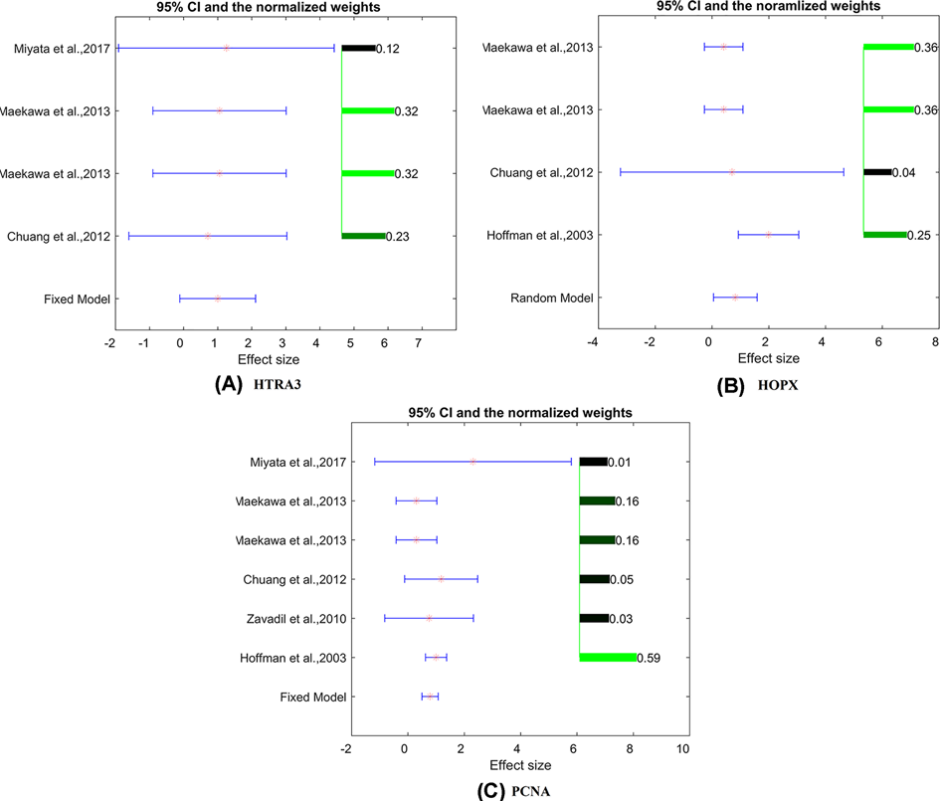
The results of the three EC inhibitors in the meta-analysis (**A**) *HTRA3*; (**B**) *HOPX*; (**C**) *PCNA*. The bar plot on the right of each figure represents the normalized weights for each dataset/study, ranged within (0, 1); the brighter (green) the color, the bigger the weight (labeled right next to the bar). The star (in red) and lines (in blue) on the left are the mean of effect size (log fold change) and 95% CI of each dataset/study, respectively.

On the other hand, for the EC negative regulators (83 genes; see UF_EC: EC_Negative), we identified three genes that were significantly overexpressed (*HTRA3, HOPX*, and *PCNA*), as shown in Table 3. These results indicate that, in the six UF studies employed here, UF activated genes that could depress EC. Figure 1 presents the effect size, 95% CI, and study weights of these three genes in the meta-analysis.

**Table 3:** Results of meta-analysis and MLR analysis

Gene name	Meta-analysis results				MLR results		
	Random-effects model	#Study	LFC	*P*-value	#Sample	Country	Study date
*HTRA3*	0	4	1.00	3.86e-2	0.00	0.00	1.00
*HOPX*	1	4	0.82	1.77e-2	1.00	0.00	0.00
*PCNA*	0	6	0.79	4.50e-8	2.32e-2	3.77e-2	0.96
*PPARG*	0	4	−1.24	4.96e-3	1.00	0.00	0.00
*EFEMP1*	0	5	−2.14	8.44e-13	1.00	4.73e-9	1.17e-7

To note, there were also two genes (*PPARG* and *EFEMP1*) that presented significantly low expression levels. Nevertheless, down-regulation of these two genes, which are implicated as negative regulators of EC, may not contribute to the development of EC. Also noted, the sample region (country) was a significant influence factor for the expression level of all the five genes, but sample size and study data were not significant for all genes, as shown in Table 3, the MLR results.

### Shared genetic basis and pathway analysis results

**Figure 2:**
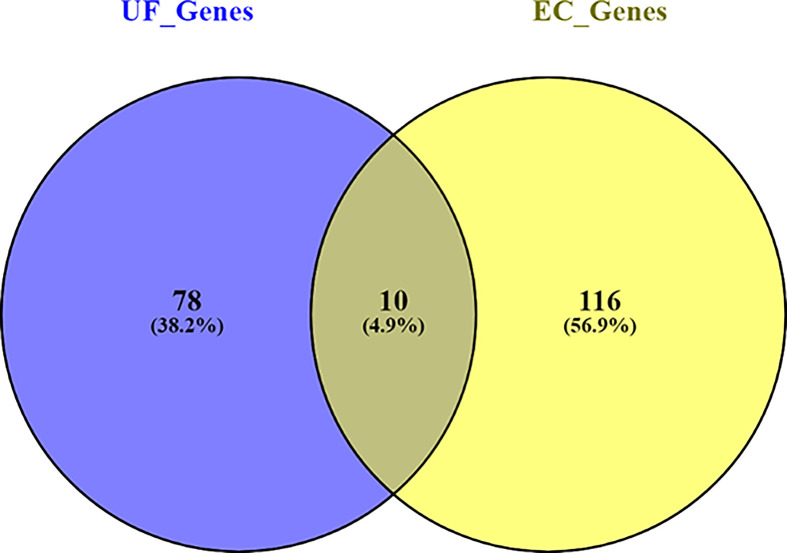
Venn diagram of the shared genetic basis by UF and EC The genes were downstream targets of UF and upstream regulators of EC that were identified by using Pathway Studio (www.pathwaystudio.com).

To explore the relationship between UF and EC at the genetic level, we used Pathway Studio (www.pathwaystudio.com) to identify and compare genes related to UF and EC with polarity, and used Fisher-exact test (https://david.ncifcrf.gov/content.jsp?file=functional_annotation.html) to explore the significance of the overlap, as shown in Figure 2. Results showed that genes linked to UF and EC presented significant overlap (*P*-value = 1.11E-10), supporting the potential relationship between UF and EC.

**Figure 3:**
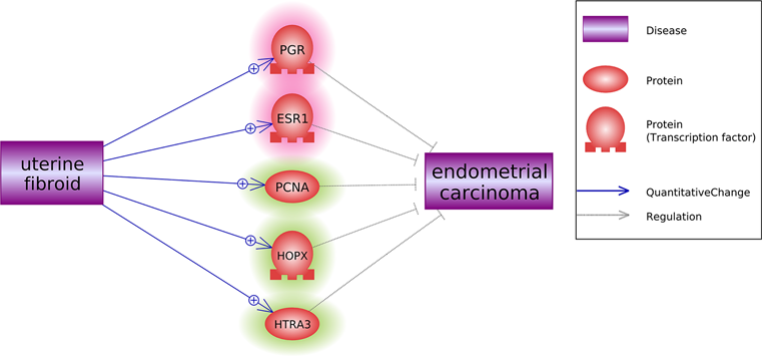
Potential pathway—the UF acts as a protective factor for EC The pathway was generated using literature-based knowledge as well as the meta-analysis results in the present study, as detailed in the supplementary data UF_EC (UF_EC_Pathway). Pathways composed of genes highlighted in red (*PGR* and *ESR1*) were these built based-on literature analyses only; pathways composed of genes highlighted in green (*PCNA, HOPX*, and *XTRA3*) were based-on both meta-analysis and literature-based pathways analysis.

Pathway Studio-guided pathway analysis [11], together with the meta-analysis results, revealed multiple potential pathways through which UF plays a role protecting against the development of EC, as shown in Figure 3.

The relation between UF and the three genes (*PNCA, HOPX*, and *HTRA3*) supported by the meta-analysis results were confirmed in the pathway analysis. The rest of the relationships (edge) presented in Figure 3 was support by multiple references, which was presented in UF_EC (UF_EC_Pathway). There were five top genes reported as regulation factors associated with EC. Among them, the expression of *PNCA, HOPX*, and *HTRA3* are additionally reported to be reduced in EC, respectively [12–15], which further suggests their negative regulator role in EC. Besides the meta-analysis results, *PGR* and *ESR1* are reported to be overexpressed in UF samples by Pathway studio [16,17] and inhibit the growth of EC simultaneously [18,19].

## Discussion

Considering the high incidence rate of UF (up to 80% of women by the age of 50) [1], it is of much interest to study the influence of UF on the incidence rate of EC. It has been stated by the U.S. Department of Health and Human Services that having fibroids does not increase the risk of developing a cancerous fibroid or getting other forms of cancer in the uterus [20]. However, one study [6] suggested that UF could increase the risk of EC in black women. In the present study, we presented a large cohort of genetic studies following the clinical data analysis of the Chinese population to evaluate the co-incidence UF and EC. Among women with gynecological diseases, the incidence rate of EC patients with a history of UF is 50.53% lower than patients without UF, suggesting a protective role of UF on EC.

Meta-analysis showed that the EC-inhibiting genes (*PCNA, HOPX*, and *HTRA3*) presented increased expression levels in the case of UF. Previous studies also showed that the expression levels of these genes were reduced in EC [12–15]. Therefore, by inhibiting these genetic markers of EC, UF could play a preventive role before the development of EC. PCNA was reported to be significantly correlated with non-endometrioid histology [13]. HOPX has been demonstrated to be down-regulated due to its promoter hypermethylation and acts as a tumor suppressor in several cancers, including endometrial cancer [15]. A study has reported that HTRA3 expression was reduced in endometrial hyperplasia as well as endometrial cancer [14], which suggests the overexpression of *HTRA3* may play a protective role in EC.

In addition to the three genes confirmed by both meta-analysis and Pathway Studio’s literature search, pathway analysis also reveals two additional genes (*PGR* and *ESR1*) that bridge UF to EC. *PGR* is reported to be overexpressed in UF samples [16], and the loss of *PGR* expression may contribute to the development of endometrial cancer as well as resistance to hormonal therapy [19]. For *ESR1*, Western blot and real-time quantitative polymerase chain reaction results showed that it was overexpressed in uterine leiomyomas [17]. On the other hand, the estrogen receptor overexpression is shown to inhibit growth and angiogenic factors in the EC cell line [18]. However, these two genes did not show an increased expression level in the six UF datasets employed in the present study. Therefore, the functionality of these two genes needs to be further tested.

Previous studies also showed that the dominant-negative mutation of rs28934576 promoted the invasive and migratory abilities of EC (10.3892/ijo.2019.4681). The mutant of rs28934576 has been shown to modulate PCNA protein levels (PMID: 28818333) and ESR1 transcription (10.1172/JCI73743). This provided further evidence for the linkage between EC and gene PCNA and ESR1. MLR analysis showed that EC was not significantly associated with other gynecological diseases in the clinical data collected in thie present study (see Table 2), which provided a clear background for the EC–UF relation analysis. However, we noticed that both EC and UF were positively related to aging.

The present study has the following limitations. First, although the sample size of the clinical data is large (4537), there are only 59 EC patients. Second, all samples were women with gynecologic diseases. Therefore, further study with a larger number of EC patients and healthy controls should be tested to confirm the conclusion of the present study.

## Conclusion

We reported a potential protective effect of UF against EC in Chinese women with gynecological diseases. The protective role of EC may be through the promotion of multiple EC genetic inhibitors, including *PCNA, HOPX, HTRA3, PGR*, and *ESR1*. Further study was guaranteed to test the genetic mechanism regarding how UF could influence EC.

## Data Availability

The datasets used and/or analyzed during the current study are available from the corresponding author on reasonable request.

## Abbreviations

CI: confidence interval
EC: endometrial carcinoma
LFC: log fold change of expression level
MLR: multiple linear regression
UF: uterine fibroid
